# Porcine reproductive and respiratory syndrome virus (PRRSV) in GB pig herds: farm characteristics associated with heterogeneity in seroprevalence

**DOI:** 10.1186/1746-6148-4-48

**Published:** 2008-11-28

**Authors:** Charlotte M Evans, Graham F Medley, Laura E Green

**Affiliations:** 1 Department of Biological Sciences, University of Warwick, Coventry, CV4 7AL, UK

## Abstract

**Background:**

The between- and within-herd variability of porcine reproductive and respiratory syndrome virus (PRRSV) antibodies were investigated in a cross-sectional study of 103 British pig herds conducted 2003–2004. Fifty pigs from each farm were tested for anti-PRRSV antibodies using ELISA. A binomial logistic model was used to investigate management risks for farms with and without pigs with PRRSV antibodies and multilevel statistical models were used to investigate variability in pigs' log ELISA IRPC (relative index × 100) in positive herds.

**Results:**

Thirty-five herds (34.0%) were seronegative, 41 (39.8%) were seropositive and 27 (26.2%) were vaccinated. Herds were more likely to be seronegative if they had < 250 sows (OR 3.86 (95% CI 1.46, 10.19)) and if the nearest pig herd was ≥ 2 miles away (OR 3.42 (95% CI 1.29, 9.12)). The mean log IRPC in seropositive herds was 3.02 (range, 0.83 – 5.58). Sixteen seropositive herds had only seropositive adult pigs. In these herds, pigs had -0.06 (95% CI -0.10, -0.01) lower log IRPC for every mile increase in distance to the nearest pig unit, and -0.56 (95% CI -1.02, -0.10) lower log IRPC when quarantine facilities were present. For 25 herds with seropositive young stock and adults, lower log IRPC were associated with isolating purchased stock for ≥ 6 days (coefficient -0.46, 95% CI -0.81, -0.11), requesting ≥ 48 hours 'pig-free time' from humans (coefficient -0.44, 95% CI -0.79, -0.10) and purchasing gilts (coefficient -0.61, 95% CI -0.92, -0.29).

**Conclusion:**

These patterns are consistent with PRRSV failing to persist indefinitely on some infected farms, with fadeout more likely in smaller herds with little/no reintroduction of infectious stock. Persistence of infection may be associated with large herds in pig-dense regions with repeated reintroduction.

## Background

Porcine Reproductive and Respiratory Syndrome (PRRS) caused by PRRS virus (PRRSV), was first reported in North America in 1987 and in the United Kingdom in 1991 [1]. Current estimates are that 79% of breeder to finisher units in the UK are affected with PRRSV or are using vaccination (National Animal Disease Information Service, UK, 2007). The disease causes significant economic losses to the pig industry, costing approximately $560 million per year in the United States alone [2].

The clinical signs of PRRSV are reproductive loss in sows including return to oestrus, abortion, premature farrowing, mummified foetuses and stillbirths [3,4]. PRRSV causes high pre-weaning mortality in piglets infected *in utero *[5] and immunosuppression and consequent increase in susceptibility to other infectious diseases, particularly respiratory diseases in pigs infected post-weaning [6]. The clinical disease caused by PRRSV is highly variable between farms. For example, whilst some seropositive herds have fairly consistent rates of respiratory disease [7,8], others have periodic outbreaks of reproductive disease in breeding sows [9] suggesting that the virus does not behave consistently between farms. There has also been a report of natural fadeout of PRRSV on a farm [10] and some reports of active elimination of PRRSV from individual herds [11,9,13].

The role of fadeout and persistence in determining viral transmission dynamics has been recognised for some time, especially in the context of measles and other childhood infections [14,15]. Periodic outbreaks of measles (and therefore episodes of fadeout) have been observed in small communities [14], with low rate of supply of susceptible individuals (births) and low rates of virus introduction [15]. Persistence of a virus in a host population is critically determined by the availability (proportion) of susceptibles in the population, which is determined by, *inter alia*, transmissibility of the virus, infectious period and existence of alternative hosts or environment contamination [16-18]. Thus, for PRRSV, the observed variable clinical signs and natural fadeout might occur because of variability in virus transmission within and between farms, different strains of virus, and/or because of transmission dynamic heterogeneity that results when most of the herd becomes immune.

Anti-PRRSV antibodies (detectable by ELISA) arise approximately 9 – 13 days after infection [19] and decay over time [19,12], persisting for up to 28 months [12]. Most pigs clear virus within 3–4 months of exposure [20], so most PRRSV antibody positive pigs are virus negative and consequently seropositivity is an indicator of past infection or vaccination. Whereas seropositivity of adult pigs might have been acquired many months previously in a herd in which the virus has become absent, seropositivity of young stock born on a farm indicates virus presence on that farm.

In this paper we present the farm and pig characteristics associated with herd seropositivity and pig heterogeneity in seroprevalence to PRRSV on 103 GB pig herds using ELISA antibodies as a marker for previous exposure to PRRSV and hypothesize on patterns of fadeout and persistence.

## Methods

### Study population and data collection

Data used in this study came from a cross-sectional study of 103 pig herds in England, Wales and Scotland. Data were collected from June 2003 to August 2004 as part of a study of post weaning multisystemic wasting syndrome [21]. From each herd, 50 blood samples were collected: ten from pigs of both eight and 14 weeks of age and five from maiden gilts (breeding females in their first gestation) and five sows each of parity one, two, three, four and five or older. Pigs of the same age were randomly selected from the same pen. Where there were insufficient numbers of pigs, those in adjacent pens were randomly selected. The serum was removed from the whole blood by centrifugation and stored at -20°C. The sera were tested for PRRSV antibodies at Leeds Veterinary Laboratory using the CIVTEST PRRS E/S SUIS (Hipra, Girona, Spain), a commercially available indirect Enzyme-Linked Immunosorbent Assay (ELISA) with a sensitivity and specificity of 90.6% and 98.3% respectively according to the manufacturer. All tests were performed according to the manufacturer's instructions and results based on the IRPC (relative index × 100) of the sample with a cut off of ≥ 20 determining seropositivity, where:

$$\text{IRPC}=\left[\frac{{\text{OD}}_{\text{450}}\text{ Sample}-{\text{Mean OD}}_{\text{450}}\text{ Negative Control}}{{\text{Mean OD}}_{\text{450}}\text{ Positive Control}-{\text{Mean OD}}_{\text{450}}\text{ Negative Control}}\right]\times \text{100}$$

During the farm visit, the farmer was interviewed and management variables relating to the unit were recorded. Variables that were selected for use in the current analysis were plausibly associated with infectious disease transmission. These included the size and purpose of the herd, purchase of stock, quarantine facilities, biosecurity within the herd, and characteristics of the nearest pig unit (Table 1). In addition, the farmer's veterinarian completed a self administered questionnaire that included information on whether clinical signs of PRRS had ever been seen and if confirmed on the unit, when they were last seen, whether the veterinarian thought that the virus was still on the unit and whether pigs were vaccinated against PRRSV.

**Table 1:** Explanatory variables investigated in the statistical models obtained from the questionnaires with unit managers during farm visits June 2003 – August 2004

Herd attributes	Indoor or outdoor unit
	Nucleus or commercial unit
	Finisher site
	Number of sows (median 327, range 20 – 2300)
	Attending veterinarian specialist pig veterinarian
	Multiple site herd
	Pigs ever moved between sites
	Different system for sick pigs
	Sick pigs ever moved back to original batch group
	Purchased gilts mixed with sows
	Time after purchasing that gilts are mixed with sow group (median 3.5 days, range 0 – 16)
	Presence of separate gilt housing
	Mixing of pigs with different batches
Purchased stock	Purchase gilts
	Purchase boars
	Purchase semen
Biosecurity	Presence of quarantine facilities
	Quarantine facilities on- or off-site
	Period of time incoming stock are isolated (median 6 days, range 0 – 28)
	Isolated stock exposed to other pigs in the herd
	Isolated stock tested for disease
	Semen tested for disease
	Protective clothing worn by employees
	Pig-free time and who this applies to (median 48 hrs, range 0 – 168)
	Footdip onto the unit and who this applies to
	Parking of vehicles on- or off-site
	Presence of a wheel dip onto the unit
Characteristics of nearest pig unit	Proximity of nearest pig unit (median 2 miles, range 0.1 – 17)
	Nearest pig unit indoor or outdoor unit
	Nearest pig unit nucleus or commercial unit
	Nearest pig unit finisher site
	Number of sows on nearest pig unit (median 250 sows, range 0 – 2000)
Rodents	Birds observed in pig housing
	Rodents observed in pig housing

### Data analysis

#### Seropositive pig

A pig was defined as seropositive if the IRPC of the sample was ≥ 20 units (according to manufacturer's instructions).

#### Seropositive herd

A herd was defined as seropositive if at least one pig in the herd was seropositive. Given the specificity of the ELISA (98.3%), a sample size of 50 pigs from a disease free population would result in less than one false positive pig being detected (mean 0.85). This definition minimises the false negative errors.

#### Vaccinated herd

A herd that, according to the veterinarian, was vaccinated. If the veterinarian did not give a response regarding vaccination, it was assumed that vaccination was not used.

FreeCalc (Version 2)  was used to calculate the minimum expected seroprevalence on a farm and by age when no seropositive animals were detected, adjusted by the sensitivity and specificity of the ELISA.

The total proportion of pigs seropositive per farm and for each age category was calculated and vaccinated and unvaccinated seropositive herds were compared. Seropositive herds were categorised according to whether there were any seropositive eight and 14 week old pigs (young stock) on the unit or not. The veterinarian questionnaire results were used to investigate the history of PRRS on all herds.

### Statistical modelling

Three models were built.

Model 1: A binomial logistic regression model was used to determine associations between farm characteristics and the probability that a herd was seronegative for PRRSV antibodies. All veterinarians of vaccinated herds stated that PRRS had been seen on the units, so both vaccinated and positive herds were included in the model as seropositive herds. Analysis was carried out in Stata SE 9.0 (Stata Corporation, College Station, Texas).

Model 2 A three level mixed normal model was built in MLwiN version 2.1 [22] to investigate the associations between quantitative IRPC values and herd-level predictor variables in seropositive herds, but where young stock were seronegative. The outcome was log (IRPC + 12) (12 was added to make all log values positive) and pig by pen by farm as the three clustered levels of the hierarchy. The fixed effects included age and management practices. The model took the form:

*Log *(*ELISApositivity *+ 12)_*ijk *_= *β*X_0 _+ *β*X_*k *_+ *β*X_*jk *_+ *ν*_*k *_+ *υ*_*jk *_+ *e*_*ijk*_

Where *β*X_0 _is the intercept and *β*X is a series of fixed effect vectors that varied at the herd (k) and pen (j). *ν*_*k *_is the variance at herd level, *υ*_*jk *_is the variance at the pen level and *e*_*ijk *_is the variance of the log IRPC between pigs.

Model 3 Is as Model 2, but includes those farms where young stock were seropositive.

For all models continuous predictor variables were investigated for linearity with the outcome variable using five quintiles. The variable was transformed into a categorical variable if the relationship was not linear by eye. Pairwise correlated variables were identified using Pearson's pairwise correlation coefficient (for continuous variables) and *χ*^2 ^tests (for categorical variables), with Fisher's exact test where appropriate. To reduce the number of predictor variables for consideration in the multivariable models, significance for the univariable screening of variables was set at P < 0.1 [23]. Forward stepwise inclusion was used to build the multivariable models and confounding was assessed by evaluating the effect of the addition and removal of variables from the models. The significance probability for the multivariable models was P < 0.05. The model assumptions were investigated by observing distributions of pig standardized residuals; pigs that had significant influence on the model were determined by observing leverage values. Using pig-level seropositivity (absence or presence of antibodies) as a comparative outcome variable to log IRPC of pigs, the models were rerun with a binomial outcome and odds ratios were estimated for Models 2 and 3.

## Results

A total of 4852 pigs from 103 herds were used in the analysis. Thirty five (34.0%) herds did not have any seropositive pigs, 41 (39.8%) had at least one seropositive pig and 27 (26.2%) were using vaccination (Table 2). The median herd size was 327 sows (range 20, 2300). Based on 10 piglets born/sow/litter, two litters produced/year and an average slaughter age of 6 months (BPEX pig yearbook, 2006), approx. 3270 rearing pigs and 327 sows would be present on a median sized farm at any one time. If one or more seropositive pigs were detected from 50 pigs that were sampled per farm, the probability of the herd being truly seropositive is 95% at a prevalence of at least 12.2% (based on the sensitivity and specificity of the test).

**Table 2:** Number of negative, vaccinated and positive herds and pigs in the study (4852 pigs from 103 herds in GB)

**Age of pigs**	**Negative**		**Vaccinated**		**Positive**		**Total**	
	Herds	Pigs	Herds	Pigs	Herds	Pigs	Herds	Pigs
8 weeks	35	348	27	264	40	395	102	1007
14 weeks	34	339	27	256	40	396	101	991
Gilts	34	166	27	135	41	204	102	505
Parity 1	33	151	26	129	41	192	100	472
Parity 2	33	157	26	131	41	193	100	481
Parity 3	32	147	25	120	39	192	96	459
Parity 4	32	142	26	123	40	192	98	457
Parity 5+	32	160	26	126	40	194	98	480
**Total**	35	1610	27	1284	41	1958	103	4852

**Figure 1:**
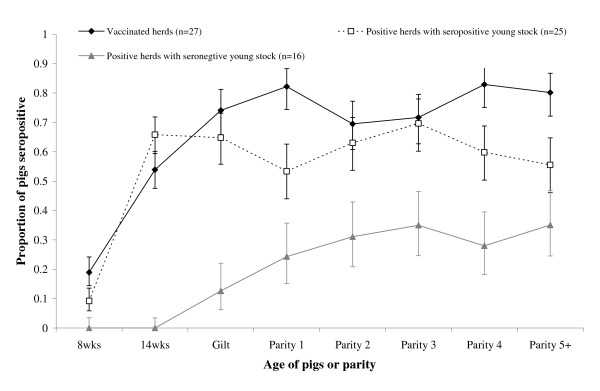
**Proportion of pigs seropositive by age for 25 positive herds that had seropositive young stock, 16 positive herds that had seronegative young stock and 27 vaccinated herds**. Bars indicate exact 95% confidence intervals. Lines are included for ease of visual interpretation only.

Vaccinated herds had a slightly higher seropositivity in adults and a similar seropositivity to 14 week old pigs in unvaccinated seropositive herds (Figure 1). Both vaccinated and unvaccinated positive herds had a significantly higher proportion of seropositive adults than herds with no seropositive young stock.

**Figure 2:**
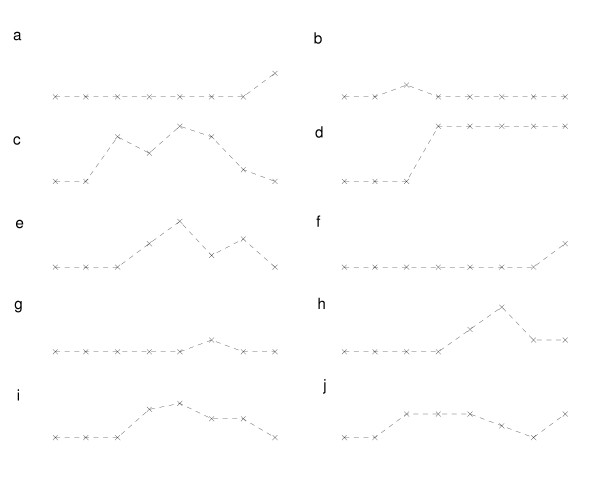
**Proportion of pigs seropositive by age for ten seropositive herds that had completely seronegative young stock and purchased replacement gilts**. x axis = Age of pigs or parity of sow (from left to right: 8 weeks, 14 weeks, gilts, parity 1, parity 2, parity 3, parity 4, parity 5+), y axis = Proportion of pigs seropositive.

**Figure 3:**
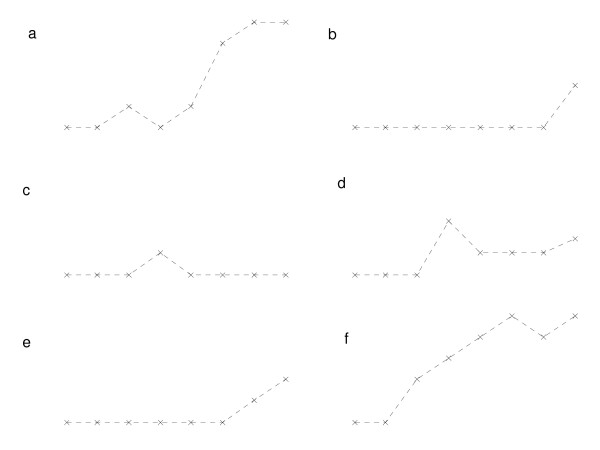
**Proportion of pigs seropositive by age for six seropositive herds that had completely seronegative young stock and only used homebred gilts**. x axis = Age of pigs or parity of sow (from left to right: 8 weeks, 14 weeks, gilts, parity 1, parity 2, parity 3, parity 4, parity 5+), y axis = Proportion of pigs seropositive.

There were sixteen positive herds that had seronegative young stock (Figures 1, 2 and 3). A herd with an average of 3270 rearing pigs would have approximately 136 pigs of each week of age from 1–24. The total number of eight and 14 week old pigs would therefore constitute 272 of the 3270 rearing pigs. A sample of 20 pigs from these age groups would be sufficient to detect a minimum seroprevalence of 22% with 95% confidence, given the sensitivity and specificity of the ELISA.

For the sixteen positive herds that had seronegative young stock, different serological patterns were observed according to whether herds purchased gilts (10 herds – Figure 2) or only used homebred replacements (6 herds – Figure 3). For herds that used homebred replacements, older sows were more likely to be seropositive compared with younger sows (Figure 3). Two out of ten herds that purchased gilts also had this pattern (Figure 2a, 2f) but for the majority of herds the seroprevalence was higher in younger sows on the farm (those purchased most recently) (Figure 2b–2e, 2g–2j) These individual farm age-seroprevalence curves demonstrate the between herd variability in exposure of pigs to PRRSV. There were no seropositive herds that had positive young stock and negative adults, nor were there more seropositive 8 week old pigs compared to 14 week old pigs in any herds.

For 33 seronegative herds for which there were veterinarian responses, 26 reported never having seen PRRS on the unit and four stated that the disease had been seen, three of which had been confirmed positive by laboratory diagnosis. All veterinarians of the 27 vaccinated herds stated that PRRS had been seen on the unit in the past and 24 reported that PRRSV was still present. Of 25 seropositive herds with seropositive young stock, 21 veterinarians reported that they had seen PRRS in the past and 12 reported clinical signs since 2000. In addition, of sixteen seropositive herds that had negative young stock, seven attending veterinarians had seen PRRS on these farms in the past and five reported clinical signs since 2002.

In the binomial logistic regression model (Model 1, Table 3), herds were more likely to be seronegative for PRRSV antibodies if there were < 250 sows on the unit (OR = 3.86, 95% CI 1.46, 10.19) and if the nearest pig unit was situated more than 2 miles from the index herd (OR = 3.42, 95% CI 1.29, 9.12). A herd size of < 250 sows was correlated with the unit being a nucleus or multiplier unit rather than a commercial unit. However, there were no other significant differences between commercial and nucleus or multiplier herds; therefore, the number of sows was included in the model. The nearest unit > 2 miles away was correlated with the nearest unit not being a commercial unit (it was a nucleus, multiplier unit, hobby farm or an isolation unit).

**Table 3:** Model 1.

**Variable**	**Sample size**	**OR**	**SE**	**p**	**95% CI**	
**Herd size**						
≥ 250 sows	75	reference				
< 250 sows	27	3.86	1.91	< 0.05	1.46	10.19
**Distance to nearest pig herd**						
< 2 miles	40	reference				
≥ 2 miles	62	3.42	1.71	< 0.05	1.29	9.12

Multivariable logistic regression model of factors associated with herds that were negative for PRRSV antibodies compared to those seropositive or vaccinated (103 herds in total).

Odds ratio (OR); standard error of the odds ratio (SE); probability function (p); 95% confidence interval (CI) (exp(coefficient+/-(1.96 × standard error)))

The mean log IRPC for pigs in seropositive herds was 3.02 (range, 0.83 – 5.58). In both Models 2 and 3, the log IRPC of pigs changed with age (Tables 4 and 5). In Model 2 (farms with seronegative young stock), the mean pig IRPC was 0.56 units lower when there were quarantine facilities on farm (95% CI -1.02, -0.10) and for every increasing mile distance between pig units there was a reduction in the log IRPC of 0.06 (95% CI -0.10, -0.01) (Table 4). The addition of the fixed effects accounted for 51.5% of herd-level variability. In Model 3 (farms with seropositive young stock), the mean pig IRPC was 0.61 units lower in herds that purchased gilts rather than used homebred replacements (95% CI -0.92, -0.29), 0.46 units lower when the farmer isolated incoming stock for six days or more (95% CI -0.81, -0.11) and 0.44 units lower if the statutory pig free time for visitors was at least 48 hours (95% CI -0.79, -0.10) (Table 5). The addition of fixed effects accounted for 64.8% of all herd-level variability. The model fit was good for both Models 2 and 3 and the assumptions of normality were reasonable. Variables significant in the final multilevel models were also significant when the binomial outcome variable (seropositive/seronegative) was used instead of pigs' log ELISA IRPC (p < 0.05) (Tables 4 and 5).

**Table 4:** Model 2.

**Variable**	**n**	**Coefficient**	**SE**	**p**	**CI^a^**		**OR**	**CI^b^**	
**Intercept**		2.48	0.25						
**Age category**									
8 weeks		reference					reference		
14 weeks	16	-0.06	0.2	0.76	-0.45	0.33	reference		
Gilts	16	0.52	0.2	0.01	0.12	0.92	reference		
Parity 1	16	0.93	0.2	< 0.01	0.53	1.33	10.8	3.54	33.02
Parity 2	16	1.14	0.21	< 0.01	0.74	1.54	16.3	5.44	48.84
Parity 3	16	1.19	0.2	< 0.01	0.79	1.59	18.8	6.4	54.88
Parity 4	16	1.08	0.2	< 0.01	0.68	1.48	12.72	4.21	38.41
Parity 5+	16	1.31	0.2	< 0.01	0.91	1.71	8.39	2.87	24.56
**Distance to nearest pig unit (miles)**	16	-0.06	0.02	< 0.01	-0.1	-0.01	0.88	0.78	0.99
**Quarantine facilities on farm**									
Not present	4	reference							
Present	12	-0.56	0.24	< 0.05	-1.02	-0.1	0.27	0.08	0.87
**Estimation of random effects:**									
Variation between herds		0.12	0.06						
Variation between pens		0.29	0.05						
Variation between pigs		0.24	0.01						

Multivariable three level mixed model of factors associated with log IRPC of 774 pigs belonging to 16 herds that had seronegative young stock.

Sample size (n); standard error (SE); probability function (p); 95% confidence interval (CI^a^) when pigs' log IRPC (+12) values were used as a continuous outcome variable; Odds Ratios (OR) and 95% confidence interval (CI^b^) when pig-level seropositivity (absence/presence of antibodies) was used as a comparative binary outcome variable (exp(coefficient+/-(1.96 × standard error))).

**Table 5:** Model 3.

**Variable**	**n**	**Coefficient**	**SE**	**p**	**CI^a^**		**OR**	**CI^b^**	
**Intercept**		2.85	0.16						
**Age category**									
8 weeks	24	reference							
14 weeks	24	1.47	0.14	< 0.01	1.19	1.75	23.57	11.32	49.06
Gilts	25	1.52	0.15	< 0.01	1.23	1.81	26.31	11.99	57.74
Parity 1	25	1.36	0.15	< 0.01	1.07	1.65	13.37	6.14	29.11
Parity 2	25	1.52	0.15	< 0.01	1.22	1.81	22.81	10.39	50.05
Parity 3	24	1.6	0.15	< 0.01	1.3	1.9	29.93	13.32	67.25
Parity 4	24	1.38	0.15	< 0.01	1.09	1.68	17.98	8.21	39.37
Parity 5+	24	1.42	0.15	< 0.01	1.13	1.71	14.73	6.75	32.14
**Purchased gilts**									
No	13								
Yes	12	-0.61	0.16	< 0.01	-0.92	-0.29	0.35	0.18	0.7
**Length of time purchased stock isolated**									
Not isolated	14	reference							
1–5 days	3	-0.36	0.25	0.14	-0.85	0.12	0.61	0.21	1.77
6 days or more	8	-0.46	0.18	< 0.01	-0.81	-0.11	0.43	0.2	0.93
**Pig free time for visitors**									
< 48 hours	17	reference							
≥ 48 hours	8	-0.44	0.18	< 0.05	-0.79	-0.1	0.44	0.2	0.93
**Estimation of random effects:**									
Variation between herds		0.11	0.04						
Variation between pens		0.2	0.03						
Variation between pigs		0.45	0.02						

Multivariable three level mixed model of factors associated with log IRPC of 1184 pigs belonging to 25 herds that had seropositive young stock.

Sample size (n); standard error (SE); probability function (p); 95% confidence interval (CI^a^) when pigs' log IRPC (+12) values were used as a continuous outcome variable; Odds Ratios (OR) and 95% confidence interval (CI^b^) when pig-level seropositivity (absence/presence of antibodies) was used as a comparative binary outcome variable (exp(coefficient+/-(1.96 × standard error))).

## Discussion

The 103 herds sampled in this study were representative of the national herd in size, location and ratio of indoor to outdoor pig herds in 2004 [21]. Age-related antibody profiles were heterogeneous between farms, and much of the heterogeneity is explained by covariates that would be expected to be related to virus introduction (pig density, quarantine) or persistence (herd size). Although the data are from a cross-sectional study, the ELISA results indicate past exposure to PRRSV from which time-dependent patterns can be inferred. The prevalence of antibody positive pigs in one age group is not necessarily associated with the prevalence in another, because exposure to virus may have occurred at different times and, for sows, even on a different farm. The presence of antibodies in young stock indicates virus presence and transmission on that farm.

Classifying herds as virus negative on the basis of 20 seronegative 8 or 14 week old pigs (irrespective of whether seropositivity was non-homogeneously distributed within the two age groups) is supported by three arguments. First, there was no passive immunity, which declines within 4–10 weeks [24,25] indicating that sows were likely seronegative. Second, in the current study, unvaccinated herds with seropositive young stock had a mean seroprevalence of 67% in 14 week old pigs (Figure 1). When all 20 grower pigs were seronegative the true seroprevalence would be expected to be ≤22%, with 95% confidence. This suggests that young stock might act as sentinels for active virus transmission within a herd: they are either negative or highly positive. Third, both vaccinated and unvaccinated positive herds had a significantly higher proportion of seropositive adults than herds with no seropositive young stock, indicating that these herds had no/little active infection in adults.

The factors that may provide a pool of susceptible pigs and reduce the probability of herd immunity and so aid persistence of PRRSV on pig farms include production of susceptible piglets (approximately 22 per annum per sow) and the movement of pigs between farms, especially breeding stock, currently replaced at 45% per annum in the UK. These risks are correlated and decrease together as herd size decreases. There may be a threshold level when the probability of successful introduction reduces to below 1. In the current study, this appears to be at ~250 sows. So a smaller herd size might reflect an increased probability of reduced risk of introduction of virus and/or virus fadeout from lack of susceptible pigs. The association between fadeout of PRRSV and herd size has been reported previously [10].

Herds with < 250 sows were correlated with multiplier and nucleus herds. Such herds were also more likely to be situated > 2 miles from the next nearest pig unit. It is not possible to state which of these factors, or the combination of factors, assists seronegativity because these farms are more likely to be more biosecure than commercial farms and might be deliberately situated further away from the main pig breeding areas. Consequently, cause and effect relationships cannot be separated. However, biologically, a small population is more likely to lead to virus fade out.

PRRSV antibody negative herds were more prevalent (Model 1) when they were > 2 miles away from the nearest pig unit. Seropositivity was also lower in herds that were more remote from other pig herds so local distant spread appears possible. The mechanisms by which virus may be transmitted between herds is currently not known, although aerosol transmission of PRRSV has been demonstrated over short distances [26,27] and some birds and insects can harbour virus [28,29] and so might transmit virus over longer distances. The association of lower IRPC values in positive herds when statutory pig-free time for visitors was > 48 hours may reflect a possible route of introduction of virus, although not reported previously. It is also likely to correlate with generally high biosecurity. However the virus is transmitted, researchers in Denmark and the UK suggest that between-herd transmission of virus, not via pigs, is possible [1,30].

As well as geographical isolation, purchase of known negative stock and quarantine of stock before introduction onto a farm may limit introduction of PRRSV. Presence of quarantine facilities were not associated with antibody negative herds but were associated with herds that had all seronegative young stock (Model 2), which are likely to have been virus negative (see below). In addition, time that purchased stock spent in isolation was associated with lower IRPC values in pigs in virus positive herds (Model 3). This latter association could occur if pigs in isolation were more likely to be virus negative by the time they entered the unit. Isolation of new stock has been associated with a lower risk of introduction of PRRSV [1,31,8].

A lower mean IRPC in positive herds with purchased gilts rather than homebred replacements would occur if there was a higher probability that purchased gilts were seronegative compared with homebred gilts: this probability would be high if the mean herd seropositivity was higher than the mean of all herds. Purchase of PRRSV negative gilts into a PRRS virus positive herd might lead to disease in the herd if these gilts were infected when pregnant and this may explain some of the irregular disease patterns reported in positive herds [9].

The presence of antibodies in breeding female pigs but not young stock (Figures 2 and 3) has two possible explanations. First, gilts were exposed to virus or vaccinated on one farm and then introduced into a negative herd when they were seropositive but virus negative. Second, if virus had been transmitted on the farm in the recent past, but had since faded out, then it would leave seropositive virus negative older parity sows. This is seen in six herds that had seronegative young stock and used homebred replacement gilts (Figure 3a–3f), where older sows were more likely to be seropositive. This suggests fadeout of virus from the herd, since older pigs are more likely to have been present in the herd when virus was circulating. Following fadeout of virus, younger pigs in the herd would not have been exposed to virus and so would remain seronegative. Four of these six herds that used homebred replacements had some seropositive gilts or parity 1 sows (suggesting that virus was present up until quite recently) (Figure 3a, 3c, 3d, 3f). This may suggest either the early stages of fadeout (with younger pigs having not been exposed to virus) or the early stages of an outbreak, with virus in the breeding herd but not yet in the young stock. Two of the profiles of herds that had seronegative young stock and purchased gilts also suggest virus fadeout (Figure 2a, 2f), but the remaining eight had a higher proportion of seropositive younger sows compared with older sows (Figure 2b–2e, 2g–2j). This suggests introduction of antibody positive stock into virus negative herds and a decline in the level of antibodies in older sows because they had been purchased, and presumably exposed, a long time previously and the level of antibodies had waned. These profiles suggest non re-exposure to virus after purchase and therefore a lack of virus in the recipient herd.

It is likely that both truly virus positive and truly virus negative herds that use homebred replacements are the most clinically and immunologically stable, since the former encourages active immunity in pigs before their first gestation and the latter have no virus. Introduction of viraemic stock into positive herds might assist in persistence of PRRSV through re-introduction and would be of concern if a different strain of PRRSV was introduced. Conversely, introduction of negative pigs into a positive herd might cause disease because these gilts would be infected in their first gestation. Totally negative herds are at risk of PRRSV introduction if geographically close to another pig farm, of larger herd size or if purchasing and/or not isolating incoming stock. We are pursuing the development of mathematical models to better understand the transmission dynamics of PRRSV, the patterns of clinical disease it exhibits and appropriate control strategies.

Approximately 51.5% and 64.8% of the total between-herd variance (amongst seropositive herds) were explained in the two multilevel models (Tables 4 and 5) respectively. As a result, the proportion of variation attributable to differences between pens and between pigs was high in both multivariable models. Approximately 37% and 59% of the total variation was attributable to differences between pigs in the two models (Tables 4 and 5) respectively. The collection of data at the pen and pig levels may have accounted for some of this variability. Main sources of variability may include IRPC values between pigs (experimental error and strain variation) as well as presence of maternal antibody and the time of exposure to virus.

The decision to use ELISA log IRPC values as the continuous outcome variable in the multilevel models was based on < 100% sensitivity of PRRSV ELISAs [32,33]. A binary outcome would have led to a possible misclassification if pigs with low PRRSV IRPC values were coded as seronegative when they were, in fact, low seropositive and vice versa. The normality of residuals and the similar pattern of significance of variables present in the multivariable models when the binary outcome was used imply the suitability of the data to this type of analysis.

## Conclusion

We conclude that PRRSV infection was far from consistent across this sample of farms, with herds ranging from seropositive pigs in all age groups, to seronegative in young stock and seronegative in all ages. The results suggest that PRRSV transmission dynamics exhibit viral fadeout and reintroduction rather than indefinite persistence on infected farms. Whilst fadeout may occur in smaller more geographically isolated herds with minimal introduction of infectious stock, persistence may be associated with large herds in pig-dense regions with continuous introduction of infectious stock. These results may explain why disease is variable between infected herds and indicate that different management strategies are required which depend on the current herd status.

## Authors' contributions

CME was involved in the analysis and interpretation of the results and statistical analyses. GFM was involved in the development of the research concept and ideas. LEG designed the study, participated in its coordination and was involved in the interpretation of results and the development of the research concept and ideas. All authors contributed to the drafting of the manuscript and have read and approved the final version.
